# The suppressive effect of a commercial extract from *Durvillaea potatorum* and *Ascophyllum nodosum* on infection of broccoli by *Plasmodiophora brassicae*

**DOI:** 10.1007/s10811-015-0564-y

**Published:** 2015-03-21

**Authors:** D. Wite, S. W. Mattner, I. J. Porter, T. Arioli

**Affiliations:** 1 Department of Environment and Primary Industries, Knoxfield, Victoria Australia; 2 Victorian Strawberry Industry Certification Authority, Toolangi, Victoria Australia; 3 School of Botany, La Trobe University, Bundoora, Victoria Australia; 4 Seasol International, Bayswater, Victoria Australia

**Keywords:** Clubroot, Kelp extract, Seaweed extract, Seasol®

## Abstract

A sand solution technique demonstrated the capacity for a commercial seaweed extract from *Durvillaea potatorum* and *Ascophyllum nodosum* (Seasol Commercial®) to significantly suppress infection of broccoli by *Plasmodiophora brassicae*. In the primary stages of infection, the extract reduced the number of plasmodia formed in the root hairs by 55 %. Later, in the secondary stages of infection, the extract reduced plasmodia in the root cortical cells by up to 84 %. The suppression of infection was found to be independent of the dilution of the extract applied (1:25 and 1:500). The basis for these results is unlikely to be a nutrient or pH effect since the extract had little impact on these parameters, particularly at the lower dilution (1:200). Rather, we hypothesise that the suppression of infection by the seaweed extract was due to its stimulation of resistance mechanisms in the host, which is possibly related to laminarins in the extract and/or the effect of exogenous growth regulators or undiscovered molecules in the extract disrupting the infection process.

## Introduction

Clubroot caused by the obligate soil-borne parasite *Plasmodiophora brassicae* is considered the most important soil-borne disease of brassica crops, such as broccoli, cabbage, canola and others (Dixon 2014; Donald and Porter 2009; Donald and Porter 2014). The pathology of clubroot infection consists of several phases—primary phases that occur in the root hair and secondary phases that occur in the cortex. An amoeboid form of the pathogen may link the phases of infection (Donald et al. 2008). Ultimately, infection can result in abnormal tissue proliferation in the host and the formation of root galls, which are characteristic of the disease.

Changes in auxin and cytokinin metabolism are thought to be key mechanisms in the development and formation of clubroot galls (Devos et al. 2005; Siemens et al. 2006). Compared with healthy plants, concentrations of indole-3-acetic acid (IAA) or conjugated IAA in infected roots alternate from higher to lower levels (Kavanagh and Williams 1981; Ludwig-Müller et al. 1993; Devos et al. 2005), with the transition possibly correlating with the change from primary to secondary phases of infection (Devos et al. 2005). By comparison, most authors report increased cytokinin activity in infected compared with healthy plants (Dekhuijzen and Overeem 1971; Dekhuijzen 1980), with Müller and Hilgenberg (1986) showing that plasmodia of *P. brassicae* can synthesise the cytokinin, trans-zeatin. However, Devos et al. (2005) found that infection reduced the total active cytokinin content of root tissue and hypothesised that plasmodia of *P. brassicae* act as a sink for zeatin in the primary infection phases. In *Arabidopsis thaliana*, Siemens et al. (2006) found that genes involved in auxin homeostasis were upregulated whereas those involved in cytokinin homeostasis were downregulated during the infection process. Despite the potential role of auxins and cytokinins in the formation of clubroot galls, there are few studies on the effects of exogenous applications of these and other growth regulators on pathogenesis, especially when they are applied in seaweed extracts.

It is well documented that seaweed extracts can improve crop health when applied as soil amendments or foliar treatments in horticultural systems (Khan et al. 2009, Craigie 2010, Arioli et al. 2015). For example, Mattner et al. (2013) showed that a commercial extract from the brown algae *Durvillaea potatorum* and *Ascophyllum nodosum* reduced the incidence of white blister, caused by *Albugo candida*, in the establishment of broccoli seedlings by 23 %. Seaweed extracts can affect soil-borne diseases through a direct suppressive effect on pathogens and pathogenicity (Mattner et al. 2014) or through stimulation of antagonistic microflora in treated soil (Dixon and Walsh 2004). Most evidence, however, suggests that seaweed extracts or their components, such as laminarins, can stimulate resistance mechanisms in the host against pathogen infection (Aziz et al. 2003, Jayaraj et al. 2008, Jayaraman et al. 2010, Subramanian et al. 2011). Moreover, growth regulators contained in seaweed extracts, including auxins, cytokinins and betaines, have the potential to further moderate these resistance mechanisms (Kazan and Manners 2009; Naseem and Dandekar 2012).

In Australia, a significant number of brassica growers have applied seaweed extracts as components of their management strategies for clubroot. Despite this, few scientific studies report on the effects of seaweed extracts on infection of brassicas by *P. brassicae* or on clubroot. In pot trials, Stewart (2008) found that soil amendment with two commercial seaweed products derived from *Ascophyllum nodosum*, a *Laminaria* sp. and a *Sargassum* sp. reduced the disease severity of clubroot by up to 33 %, although this was not significant at the *p* ≤ 0.05 level. When considering the activity of commercial seaweed extracts on plant health, it is important to recognise that their effects and properties may differ due to the species of seaweed utilised and the commercial extraction systems employed in their production (Verkleij 1992). Our study used a sand solution technique to test the hypothesis that a commercial seaweed extract from *D. potatorum* and *A. nodosum* can reduce infection of broccoli by *P. brassicae*.

## Method

### Sand solution technique

The sand solution technique used in this experiment is described by Donald and Porter (2004) and is an established method to observe infection of brassicas by *P. brassicae*. In this technique, acid-washed, coarse sand (Banksia Nurseries, Knoxfield, Australia) was autoclaved (121 °C/100 kPa) and loosely packed into 5–mL tapered pipette tubes (Oxford Labware, USA). Broccoli seed (cv. Marathon, Henderson Seeds, Templestowe Lower, Australia) was surface-sterilised for 3 min in a 70 % ethanol/10 % sodium hypochlorite solution, followed by three rinses in sterile distilled water, and dried in a laminar flow cabinet overnight on sterile filter paper. A single seed was sown into the sand in each of the pipette tubes. Two tubes were placed inside a larger 50-mL Falcon tube containing a buffered nutrient solution (MES Solution, USA). This nutrient solution was developed by Myers and Campbell (1985) to maximise *P. brassicae* infection, without causing nutrient stress in the host, and consists of the following: MgSO_4_ (1 mM), KNO_3_ (2.5 mM), Ca(NO_3_)_2_ (2.5 mM), KH_2_PO_4_ (0.5 mM), H_3_BO_3_ (92 μM), MnCl_3_ (18 μM), CuSO_4_ (0.64 μM), H_2_Mo_2_O_4_ (0.14 μM), ZnSO_4_ (1.5 μM) and Fe-EDTA (1.3 μM). At 5 days after sowing, 2 mL of seaweed extract (1:25 or 1:200 dilutions, see below) or sterile distilled water for the control were applied to the sand within the pipette tubes. At 28 days after sowing, stored (−20 °C) spore suspensions of *P. brassicae* collected from infected broccoli plants in Werribee South, Victoria, Australia (ECD code 16/19/31), were thawed and resuspended in sterile distilled water to contain a concentration of 10^6^ spores mL^−1^, using a haemocytometer. Plants in the pipette tubes were inoculated by applying 0.2 mL of the spore suspension to the sand. Seedlings were kept in a growth cabinet (25 °C day, 20 °C night, 90 % relative humidity, 12-h days) for the duration of the experiment, and nutrient/seaweed extract solutions replaced as required.

### Seaweed extract

The seaweed extract used in the experiment was an alkaline hydrolysis product (Arioli et al. 2015) from *D. potatorum* and *A. nodosum* (Seasol Commercial®, Seasol International, Bayswater, Australia), which was sterilised by exposure to UV for 2 h in a laminar flow cabinet. This method has previously sterilised small volumes (15 mL) of the extract without reducing its bioactivity (Mattner et al. 2014). The undiluted extract has a pH of 10.5 and contains 0.2 % (*w*/*v*) N, 0.02 % P, 3.7 % K, 0.3 % S, 458 mg L^−1^ Ca, 972 mg L^−1^ Mg, 115 mg L^−1^ Fe, 2 mg L^−1^ Mn, 15 mg L^−1^ B, and 5 mg L^−1^ Zn (Seasol International 2015). In addition to low concentrations of nutrients, the undiluted extract contains 7 % (*w*/*v*) total laminarins, 154 μg L^−1^ total auxins, 36 μg L^−1^ total cytokinins (including zeatin, dihydrozeatin, isopentenyl-adenine, and their corresponding ribosides and glucosides; Tay et al. 1985, 1987) and 382 μg L^−1^ total betaines (Seasol International 2015). Total concentrations of nutrients and growth regulators in the sand solutions in the different treatments were determined based on these figures and the prepared concentrations of the buffered nutrient solution (see above). In addition, the pH of the sand solutions in the different treatments was measured using a pH meter.

### Infection assessment

Infection was assessed at 14, 28 and 45 days after inoculation (d.a.i.). At each assessment date, the roots of six plants per treatment (i.e. two replicate plants by three blocks) were harvested, rinsed free of sand, fixed with formyl acetic acid and then stained with FFA Phloxine B to allow observation of the development of infection by *P. brassicae*. Twenty root sections (200 μm long) per plant (i.e. 120 root sections in each treatment) were examined under compound microscope for the number of plasmodia in the root hairs and cortical cells.

### Trial design and treatments

The experiment was conducted as a randomised complete block design, with three blocks. Each block contained six replicate plants of each treatment (i.e. a total of 18 plants per treatment). Two plants per treatment per block (i.e. six plants per treatment) were harvested at each of the three sampling dates (see above). Treatments included 1:25 (plus and minus inoculation) and 1:200 (plus inoculation) dilutions of seaweed extract, and sterile distilled water (plus and minus inoculation) as the control. Data were analysed by ANOVA using Genstat v. 12.1 (VSN International, UK). Homogeneity of variance was determined from plots of fitted values versus residuals, while histograms of residuals were examined for normality of distribution. Fisher’s least significance difference test was used to identify significant differences between treatment means. The level of significance used was *p* ≤ 0.05.

## Results

Due to the low nutrient content of the undiluted seaweed extract and the dilutions used in this experiment, differences in the pH and nutrient content of the sand solutions in the different treatments were negligible, except for K and Fe (Table 1). In particular, the nutrient content between the control and the 1:200 seaweed extract treatment were similar. Unlike the seaweed extract treatments, however, the sand solution in the control contained no laminarins or growth regulators.

**Table 1:** Calculated concentration of elements and compounds in the sand solutions of different seaweed extract (SE) treatments

Component	Control	SE (1:25)	SE (1:200)
pH	5.73	6.14	5.79
N (mg L^−1^)	97.3	103.2	98.0
P (mg L^−1^)	14.3	14.9	14.4
K (mg L^−1^)	108.6	218.2	122.3
S (mg L^−1^)	29.7	38.6	30.9
Ca (mg L^−1^)	92.7	94.1	92.9
Mg (mg L^−1^)	22.5	25.4	22.9
Fe (μg L^−1^)	67.2	408.0	109.8
Mn (μg L^−1^)	915.6	921.5	916.4
B (μg L^−1^)	920.9	965.4	926.5
Zn (μg L^−1^)	90.8	105.6	92.7
Mo (μg L^−1^)	24.9	24.9	24.9
Cu (μg L^−1^)	37.6	37.6	37.6
Laminarins (mg L^−1^)	0.0	207.4	25.9
Auxins (μg L^−1^)	0.00	0.45	0.06
Cytokinins (μg L^−1^)	0.00	0.11	1.3 × 10^−2^
Betaines (μg L^−1^)	0.00	1.13	0.14

In the primary phases of infection, treatment with the seaweed extract reduced the number of plasmodia formed by *P. brassicae* in broccoli root hairs by up to 55 % compared with the control (Table 2). The effect was statistically significant at 28 d.a.i., but not at 14 d.a.i. At 45 d.a.i., in the secondary phases of infection, plasmodia had formed in cortical cells and there were up to 84 % fewer in the roots of plants in the seaweed extract treatments than in the control (Table 2). The dilution of seaweed extract (1:25 or 1:200) had no significant effect on the numbers of plasmodia formed by *P. brassicae* in root hairs or cortical cells of broccoli. No plasmodia formed in the non-inoculated treatments.

**Table 2:** Number of plasmodia (per 200 μm length of root section) in the root hairs and cortical cells of broccoli following treatment with a seaweed extract (SE) and inoculation with *Plasmodiophora brassicae*

Treatment	Plasmodia in the root hair		Plasmodia in cortical cells
	14 d.a.i.	28 d.a.i.	45 d.a.i.
Inoculated			
SE (1:25)	2.1	1.3	16.4
SE (1:200)	2.1	1.2	8.1
Control (water)	3.0	2.7	51.7
Non-inoculated			
SE (1:25)	0.0	0.0	0.0
Control (water)	0.0	0.0	0.0
LSD (*p* = 0.05)	1.7	0.6	8.9

*LSD* least significant difference where *p* = 0.05, *d.a.i.* days after inoculation

## Discussion

To our knowledge, this is the first published report of a commercial seaweed extract directly suppressing infection of broccoli by *P. brassicae*. In this study, suppression of infection was observed as fewer numbers of plasmodia in the roots of the host. The seaweed extract had a greater effect in suppressing the secondary phases of infection (plasmodia in the root cortex were reduced by up to 84 %) than the primary phases of infection (plasmodia in the root hairs were reduced by up to 55 %). The underlying biological reasons for this difference, however, could not be determined by the current study and warrant further investigation. It is possible that the extracts slowed the development of infection, or they interfered with transition from the primary to secondary phases of infection.

It is important to recognise that suppression of infection does not always translate to reduced clubroot severity or improved commercial yields of broccoli. Despite this, our unpublished field and glasshouse research conducted under conditions of low disease expression showed a trend towards reduced clubroot severity and gall sizes in broccoli treated with the seaweed extract. Similarly, Stewart (2008) found a trend towards reduced clubroot severity in broccoli plants treated with a different seaweed extract from *A. nodosum*, although this was not significant at the *p* ≤ 0.05 level. Further field and glasshouse trials conducted under higher disease pressures are needed to determine if the effect of the seaweed extract in suppressing infection of broccoli by *P. brassicae* translates to lower clubroot severity.

Infection of brassicas by *P. brassicae* is strongly influenced by pH, with infection increasing under acidic conditions (Donald and Porter 2004, 2009). Using the identical sand solution method as the current experiment, Donald and Porter (2004) found that raising the pH of the nutrient solution from 5.5 to 8.0 reduced the proportion of root hairs of broccoli infected by *P. brassicae* by 70 %. In contrast, raising the pH of the nutrient solution from 5.5 to 6.5 only reduced infection by 5 %. In the current experiment, the addition of seaweed extract at dilutions of 1:25 and 1:200 raised the pH of the nutrient solution—from pH 5.73 to 6.14 and 5.79, respectively. Given that both dilutions of seaweed extract inhibited *P. brassicae* infection to an equivalent level and the comparative results of Donald and Porter (2004), there is little evidence that the effect in the current trial was due to pH. Nonetheless, the use of alkaline seaweed extracts to modify pH in the rhizosphere of brassica crops warrants further investigation as a possible component in clubroot management.

Infection of brassicas by *P. brassicae* is also influenced by some nutrients (Donald and Porter, 2009). For example, increased levels of Ca (Donald and Porter 2004), Mg (Myers and Campbell 1985), and B (Webster and Dixon 1991) have the capacity to decrease infection. The nutrient content of the sand solution in the current trial was sufficient to prevent nutrient stress in the host plant but allow maximum expression of infection (Myers and Campbell 1985). Due to the dilutions used and their relatively low nutrient content, the addition of the seaweed extract treatments to plants in the experiment did not markedly affect nutrient concentrations in the sand solution (Table 1), except for increased levels of Fe and K. Fe is not noted to affect infection of brassicas by *P. brassicae* (Donald and Porter 2009), while increased levels of K can enhance infection rather than suppress it (Prabhu et al. 2007). This in addition to the fact that dilution of the seaweed extract did not affect its ability to suppress infection, suggesting that a direct nutrient effect from the extracts in reducing infection is highly unlikely.

Previous research has shown that the undiluted seaweed extract used in the current study can have a direct suppressive effect on the survival and growth of some pathogens (e.g. *Sclerotinia minor*) or on their pathogenicity (Mattner et al. 2014). By comparison, the extracts used in the current studies were diluted, so a direct biocidal effect against zoospores of *P. brassicae* seems unlikely. Moreover, there was no difference in the ability of high and low dilutions of the extract to suppress infection. Rather, it is hypothesised that the suppression of infection by the seaweed extract was due to its stimulation of resistance mechanisms in the host and/or the effect of exogenous growth regulators or other undiscovered molecules in the extract disrupting the infection process.

It is well established that some elicitors (e.g. salicylic acid) can increase the expression of resistance genes and nanomolar concentrations of defence compounds in *Arabidopsis thaliana* challenged by *P. brassicae* (Agarwal et al. 2011; Lovelock et al. 2013). Other experiments have detected similar gene responses in *Arabidopsi thaliana* treated with seaweed extracts from *Ascophyllum nodosum* (Jayaraj et al. 2008; Jayaraman et al. 2010). Similarly, laminarins contained in various seaweed extracts can elicit defence responses in plants (Klarzynski et al. 2000; Aziz et al. 2003). It is possible that the ability of the seaweed extract to reduce infection of broccoli by *P. brassicae* in the current study was due to a similar effect, since the diluted extracts contained laminarins (Table 1). Gene expression experiments using the *Arabidopsis thaliana*/*P. brassicae* system are needed to test this hypothesis.

Even though cytokinins and auxins play an important role in the development of clubroot, few studies have tested the application of exogenous growth regulators on infection. Dekhuijzen and Overeem (1971) demonstrated that *P. brassicae*-infected and non-infected callus tissue of turnip required different concentrations of exogenous auxins and cytokinins to support their growth. Siemens et al. (2006) found that a cytokinin overexpressing line of *Arabidopsis thaliana* was resistant to clubroot disease. In contrast, Stewart (2008) postulated that application of exogenous cytokinins would increase disease severity in brassica crops by stimulating root hair formation, which is the primary site of infection for *P. brassicae*. Furthermore, Arioli et al. (2015) reported the presence of other growth regulators, i.e. strigolactones and brassinosteroids, in the seaweed extract investigated in the current trial. The effects of growth regulators on the interaction between plant development and pathogen infection are complex (Kazan and Manners 2009; Naseem and Dandekar, 2012). More chemical characterisation and investigation of complex seaweed extracts is needed to uncover the true mode of action for the seaweed extract we found to disrupt the infection process of *P. brassicae* in broccoli.
